# Osseointegrated Implants: An Alternative Approach in Patients with Bilateral Auricular Defects due to Chemical Assault

**DOI:** 10.1155/2016/7371645

**Published:** 2016-04-20

**Authors:** Emilio Mevio, Mauro Mullace, Luca Facca, Stefano Schettini

**Affiliations:** ^1^Department of Otorhinolaryngology, Fornaroli Hospital, Via Donatore del Sangue 50, 20013 Magenta, Italy; ^2^F&S Prosthesis Workshop, Ariccia, Rome, 00072 Lazio, Italy

## Abstract

Acid attacks committed as crimes of passion are unfortunately becoming far from infrequent occurrence. The injuries sustained in such attacks mainly involve the face and trunk, with the acid causing cutaneous and subcutaneous tissue burns that can result in permanent scarring, loss of the nose or external ear, and severe visual impairment. Different surgical solutions have been proposed for reconstruction of the auricle following loss of the ear through traumatic injury or cancer or in patients with congenital defects: surgical reconstruction may involve the insertion of an autogenous rib cartilage framework or the use of a porous polymer material inserted into an expanded postauricular flap. Reconstruction with rib cartilage has given good results but requires more than one surgical step and may be associated with adverse events involving both the donor site and the recipient site, while rejections of polymeric prostheses have been reported following their insertion into expanded postauricular flaps. The use of a titanium dowel-retained silicone prosthesis, in which the dowel is anchored to the temporal bone, is a surgical possibility, indicated particularly in cases of pinna resection due to tumour or auricular scarring following traumatic injury.

## 1. Introduction

Facial injuries resulting from acid burns may be sustained in situations of war; in peacetime, on the other hand, they may be the result of accidents in the workplace or home or crimes of passion. In the latter case, the attacks, intended to disfigure the victim, are inflicted as a form of punishment and can be linked to a range of different situations: lovers' disputes, financial disagreements, family feuds, robberies, vendettas, and so on [1, 2]. Acid attacks are frequently reported in certain parts of the world, particularly in the Middle East, Asia, and North America. The largest case studies in the literature were reported by major burns care units in Jordan, Iran, and China [3–6]. In our country episodes of this kind have become more frequent in recent years and are mainly associated with problems in personal relationships. Sociologists suggest that this increase can be explained by the exposure given to cases of this kind in the media, which may encourage copycat behaviour. Moreover, a marked increase in these attacks, again attributable to phenomena of emulation, has also been reported in other countries in recent years: Mannan et al. [3] reported a 201% rise in such attacks between 1999 and 2004 in Bangladesh.

Nitric acid, muriatic acid, and sulphuric acid, which are readily available, are agents commonly used in revenge attacks. The target is usually the face, although burns to the scalp, neck, and arms are also frequent. More superficial injuries, involving the skin and dermis, can be repaired with skin grafts. Deep lesions on the other hand, especially ones involving the nasal or auricular cartilage, usually result in severe disfigurement that is more difficult to correct. In such cases, the patients could be treated with classic plastic surgery based on the use of rib cartilage for skeletal pinna reconstruction covered by skin grafts. However, application of these solutions is often severely complicated or precluded by the conditions of the residual tissue, hence the growing use of prostheses in nasal pyramid or outer ear reconstruction. Initially, these prosthesis were held in place by adhesives which, however, gave poor results in terms of stability and were often associated with skin irritations.

Now, however, there exists an excellent and innovative surgical technique that allows the fixation of ear epitheses by means of bone-anchored titanium implants [7, 8].

## 2. Material and Methods

**Figure 1:**
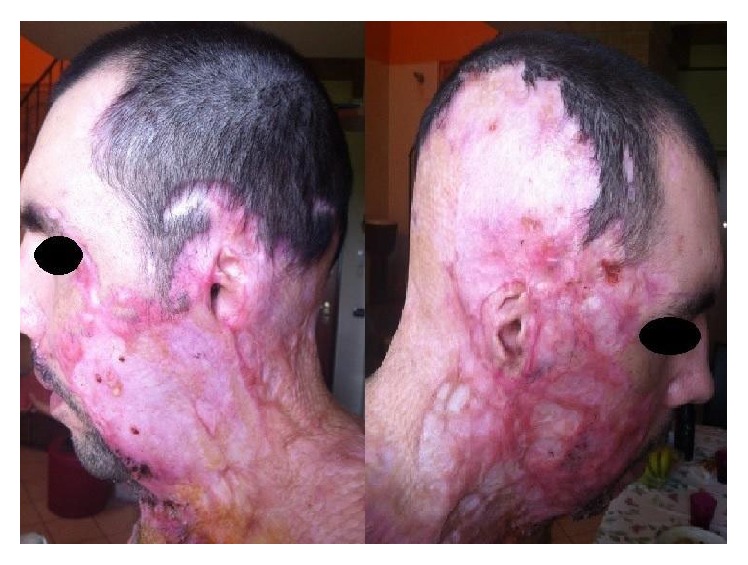
The photography of the subject before surgery. There are evidences of extensive scarring, asymmetry and deformity of face, neck, and truncus. The auricular pinnas are mutilated; the external auditory canals are intact.

The case of WP, a 38-year-old Caucasian male who, two years earlier, had been the victim of a crime-of-passion acid attack came to our attention. The attack had left him with very serious burns to the face, upper chest, and arms. In addition to suffering cutaneous and subcutaneous injuries, the patient had lost the use of his right eye (replaced with a prosthesis), while the damage to his left cornea had necessitated a transplant; he also presented with almost complete erosion of both pinnas (Figure 1). Immediately after his first hospitalisation, the patient had undergone a long series of reconstructive plastic surgery procedures involving the transplantation of autologous and nonautologous skin grafts to correct the burn injuries to the skin of his face, neck, and chest. He was referred to us to undergo prosthetic outer ear reconstruction. For three years, our department has been a reference centre in northern Italy for the implantation of auricular epitheses using the surgical technique proposed by Tjellström et al. [9]. The patient was treated using the surgical procedure described below. 

Surgical procedure: before the surgical field is prepared and with the patient's face still fully and easily visible, the implant sites should be carefully marked, using methylene blue, down to the bone. Two implants are normally sufficient for satisfactory retention. These are ideally placed approximately 20 mm from the centre of the external auditory canal opening or anticipated opening. They are positioned at 8 o'clock and 10.30 on the right side and at 4 o'clock and 1.30 on the left side.

We usually perform one-stage surgery, removing tags and remnants in cases of microtia and performing the necessary subcutaneous tissue reduction. In this patient, too, we removed the residual cartilaginous structures of the external ear, which were embedded in scar tissue (Figure 1).

An incision is made 10 mm behind the anticipated implant site. Dissection is performed down to the periosteum. A cruciate incision is then performed at each implant site. The edges are raised with a raspatory.

Drilling begins using the guide drill with the spacer kept on 3 mm. During drilling irrigation should be performed. The bottom of the hole is repeatedly checked for bone at the base of the site. If there is adequate bone thickness drilling continues to a depth of 4 mm. The drill indicator will facilitate correct drill orientation. The next step is to widen the hole to the exact diameter using a 3 or 4 mm drill countersink. Irrigation should be guaranteed.

At this point, implant installation is performed. The low speed setting should be used for implant insertion. In compact cortical bone a torque setting of 40 Ncm is recommended, whereas in soft bone a lower torque setting of 20 Ncm should be used.

The self-tapping fixture with the premounted fixture mount is seated inside the plastic ampoule in a titanium cylinder. It is then picked up with the connection to handpiece, which is placed into the drill handpiece. 

The implant is installed without cooling irrigation until the small grooves at the distal end of the implant are well within the canal. When the flange of the implant has seated the handpiece will automatically stop.

The mount is removed using the Unigrip screwdriver and the surgical wrench. The titanium standard abutment is picked up with the abutment holder and placed into the implant. We perform manual tightening, using the abutment screw, to 25 Ncm.

The skin is then repositioned over the implants. Holes are punched through the skin exactly over the abutments with a biopsy punch. The skin is then sutured. Healing caps are positioned on the abutments.

A gauze dressing is applied in a figure of 8 (foam dressing, soft silicone wound contact layer, or antiseptic dressing). The healing caps are thus held in place. The patient was discharged the day after surgery and was seen again for the first dressing after seven days. The patient received a new dressing every seven days for a month.

It is important to wait at least six weeks before loading the implants.

**Figure 2:**
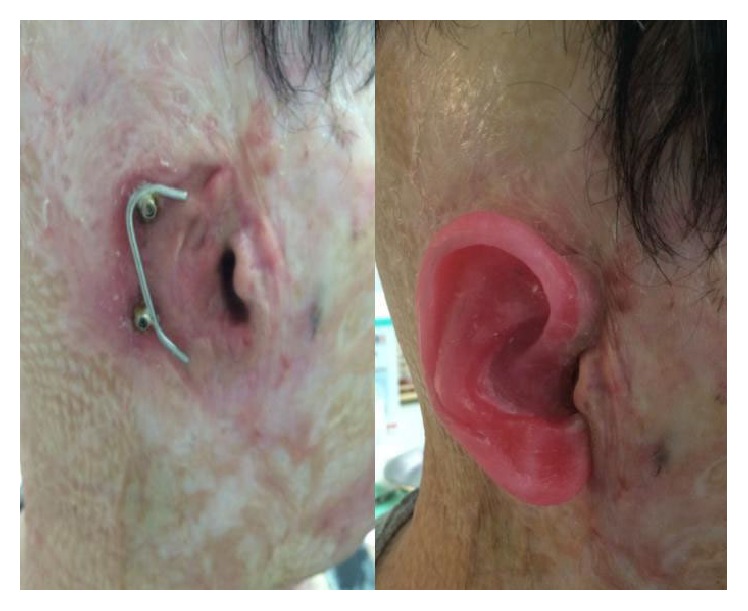
When the process of osseointegration is complete, the prostheses, which also have clips, are easily and securely attached to or removed from the gold-platinum cylinder-and-bar system.

Following healing and stabilisation of the surgical site, the patient was sent to the anaplastology technician who prepared the epitheses, modelling them on the father's pinna and carefully matching the patient's skin colour. The silicone epitheses were created using a wax pattern. The definitive ones have two sides: the inner one in an acrylic plate with clips that allow the attachment to a gold-platinum bar fixed to the abutments; the external one is made of soft silicone. When the process of osseointegration is complete, the prostheses, which also have clips, are easily and securely attached to or removed from the gold-platinum cylinder-and-bar system (Figure 2).

## 3. Discussion

The treatment of chemical burns of the face and body varies considerably depending on when the patient comes to clinical attention, the extent of the injury, and the presence of acid-induced erosion of the superficial and/or deep tissues. Indeed, the surgical management varies according to the time that elapses before the patient is seen. Immediately after the event it is easier to clean the lesion site and begin the work of reconstruction using skin flaps. However, patients often present several days (7–9 days) after the event when necrotic processes, possible superimposed bacterial infections, or the onset of spontaneous cicatrisation processes made the task of reconstruction more complicated [3].

In the case of superficial tissue damage, the lesion is cleansed, also removing any dead tissue, and it may be treated with skin flaps. Deep lesions involving the nasal pyramid or auricle, possibly severe enough to result in their partial or total amputation, are instead much more difficult to treat.

In these circumstances the usual techniques of plastic surgery are difficult to apply since they are procedures that require the presence of large areas of intact skin around the lesion (necessary for the preparation of sliding skin flaps or the use of tissue expanders prior to the placement of subcutaneous implants). This condition is not met by patients with extensive skin necrosis resulting in areas of atrophic scarring.

For this reason prostheses are being used increasingly readily. For three years, our department has been a reference centre in northern Italy for the implantation of auricular epitheses using the osseointegrated implant system, with which we have now treated 26 patients. Most of these patients were affected by microtia (*n* = 17) and several by traumatic mutilation of the external ear (*n* = 7), while the remaining ones (*n* = 2) required reconstruction following resection of the ear due to cancer. All the patients were completely satisfied with their reconstructions. No surgical complications, implant failures, or prosthetic failures were encountered.

Bone-anchored titanium implants provided these 26 patients with a safe, reliable, adhesive-free method of anchoring their auricular prostheses and restoration of their normal physical appearance.

Basically, this approach is an evolution of the implants for dental prostheses proposed by Brånemark et al. in 1969: these have been used for 40 years in the field of odontostomatology, which the same author subsequently reproposed as percutaneous craniofacial implants for use with bone conduction hearing aids [10, 11].

**Figure 3:**
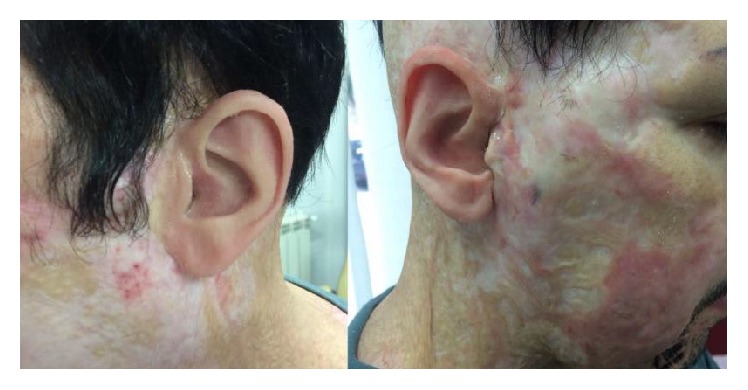
Final result: the patient after epithesis implant.

In the patient here described, who presented bilateral chemical injuries, the extent and complexity of the lesions and the considerable amount of scar tissue that had formed clearly ruled out recourse to the usual techniques of plastic surgery. We therefore opted for removal of the cartilaginous remains of the concha auriculae and tragus, followed by implantation of titanium abutments that, once properly osseointegrated, guaranteed adequate fixation of the ear epitheses. We usually treat patients with a monolateral injury and in such cases the artificial auricle is modelled on the patient's contralateral one. In this case, we decided to use the patient's father's auricle as the model. Correct osseointegration of the implants was achieved in the appropriate time and no postoperative complications occurred. The aesthetic outcome was good, as shown in Figure 3.

It should be underlined that it is crucial, also for these patients' mental and physical well-being, to try and restore to them the sense of self-dignity conferred by a harmonious physical appearance. It would be appropriate, even prior to the surgical intervention, to work out a multidisciplinary therapeutic approach for tackling the psychological and social reintegration difficulties that face patients with injuries of this kind.
